# Tracheal lesion during shoulder surgery: a case report and systemic review of the literature

**DOI:** 10.1186/s44158-021-00013-8

**Published:** 2021-10-26

**Authors:** Luigi Vetrugno, Michele Divella, Daniele Orso, Cristian Deana, Giulia Vaccher, Pietro Drovandi, Alessandro Beltrame, Araldo Causero, Tiziana Bove

**Affiliations:** 1 Department of Medicine, University of Udine, Via Colugna n. 50, 33100 Udine, Italy; 2 Anesthesia and Intensive Care Department, ASUFC University Hospital of Udine, P.le S. M. Misericordia n. 15, 33100 Udine, Italy; 3 Orthopedic and Trauma Department, ASUFC University Hospital of Udine, P.le S. M. Misericordia n. 15, 33100 Udine, Italy

**Keywords:** Arthroscopy, Pneumothorax, Pneumomediastinum, Subcutaneous emphysema, General anesthesia, Intubation, Shoulder surgery

## Abstract

Pneumomediastinum (PNM) and pneumothorax (PNX) are documented complications of arthroscopic shoulder surgery (ATS). Plexus anesthetic block and tracheal lesions during endotracheal intubation are hypothesized to be the underlying risk factors; however, the actual evidence supporting this hypothesis is scarce.

A case of bilateral laterocervical emphysema, subcutaneous edema, and signs of PNM after ATS performed under general anesthesia and supra-scapular nerve block is presented. An up-to-date systematic review of PNM/PNX during orthopedic surgery was performed, involving six databases: PubMed (1996–present), Embase (1974–present), Scopus (2004–present), SpringerLink (1950–present), Ovid Emcare (1995–present), and Google Scholar (2004–present).

Twenty-five case studies met the eligibility criteria. In 24 cases, the patient underwent general anesthesia and orotracheal intubation; in 9 of these, a plexus anesthetic block was also performed. One case involved ATS under plexus anesthetic block only. In 10 cases, the diagnostic finding was PNM. In 5 cases, the diagnostic finding was associated with PNX. PNX was detected in 17 cases. In 2 cases, SE was found in the absence of any evidence of either PNM or PNX. A tracheal lesion was identified in 3 cases.

Endotracheal intubation and loco-regional anesthesia are not the only predisposing risk factors at play in the pathogenesis of PNM/PNX. Rather, multi-factorial pathogenesis seems more probable, necessitating that specific attention is paid during ATS to the change in patient position on the operating bed, to any slipping of the endotracheal tube, to patient monitoring whilst under the drapes, and to the cuff pressure. PROSPERO registration number: CRD42021260370.

## Introduction

Pneumomediastinum (PNM) and pneumothorax (PNX) are rare but potentially fatal complications occurring during arthroscopic shoulder surgery (ATS). In the pathogenesis of PNM/PNX during ATS, three key risk factors have been hypothesized, namely (i) endotracheal intubation, (ii) interscalene brachial plexus nerve block (ISB), and (iii) arthroscopic surgery itself. For instance, some have proposed lacerations of the trachea during endotracheal intubation or lesions of the parietal pleura during anesthetic block or surgery to constitute significant risk factors of PNM/PNX [1, 2]. However, evidence corroborating their involvement is scarce, and so they continue to be considered as hypotheses only.

As these three proposed etiopathogenetic conditions are often concomitant, we performed an analysis of all case reports published in the last 30 years in order to verify the incidence of evidence of tracheal damage associated with ATS.

The aim of this review was to search the literature for all reported cases of PNM and PNX that occurred during orthopedic surgery in order to either corroborate the above-cited conditions (endotracheal intubation, ISB, and ATS) as probable risk factors or to exclude them as contributing to the pathogenesis of these complications. In addition to a literature review on this topic, a case report of tracheal rupture identified after general anesthesia for shoulder arthroscopy is presented.

## Case report

A 54-year-old female patient was scheduled for right-shoulder arthroscopy due to a lesion of the supraspinatus tendon. The patient was obese (weight: 91 kg; height 163 cm; BMI 34 kg/m^2^). No other illnesses were reported in her medical history. She was classified to have an ASA score of 2. No criteria for predicted difficult airway management were present.

Upon arrival in the operating room, standard monitoring indicated blood pressure at 140/80 mmHg, HR 70 bpm, and SpO_2_ 98% while breathing room air. A large-bore cannula (18 G) was placed in the left hand. With the patient in the sitting position, the supraspinous fossa was identified. Ultrasound long axis view with a linear probe (Esaote Mylab 25, linear probe 7.5 MHz) allowed identification of the floor of the fossa, with its characteristic “ice hockey stick” pattern. The tip of the needle (Pajunk, SonoPlex, 100 mm), aiming at the most lateral part of the scapula, was visualized using the in-plane approach. US-guided suprascapular nerve block was induced with 15 mL of levobupivacaine 0.5%. Subsequently, general anesthesia was induced with fentanyl 200 mcg, propofol 150 mg, and rocuronium 50 mg after preoxygenation with a FiO2 of 0.8. The laryngeal view obtained during laryngoscopy was classified as Cormack-Lehane grade 1. The patient was intubated using a 7.5-mm endotracheal tube with a high volume/low-pressure cuff (MALLINCKRODT Hazelwood, USA) without a rigid stylet. The tracheal tube cuff pressure was measured using an analogical manometer, and set at 20 cmH_2_O. The patient was subsequently turned on to her left side.

Anesthesia was maintained with sevoflurane at an end-tidal concentration (ET) of 1.6–1.8% and fentanyl (100 mcg). The surgical procedure lasted 1 h and 7 min. After turning the patient supine at the end of the operation, anesthetic administration was stopped to allow the patient to emerge from anesthesia. Neuromuscular transmission recovered spontaneously (evaluated with TOF-Watch, Organon, Dublin, Ireland). The woman showed signs of low tolerance to the endotracheal tube, with cough, irritability, and frequent head and neck movements; therefore, early extubation was performed after the endotracheal tube cuff had been completely deflated.

**Fig. 1:**
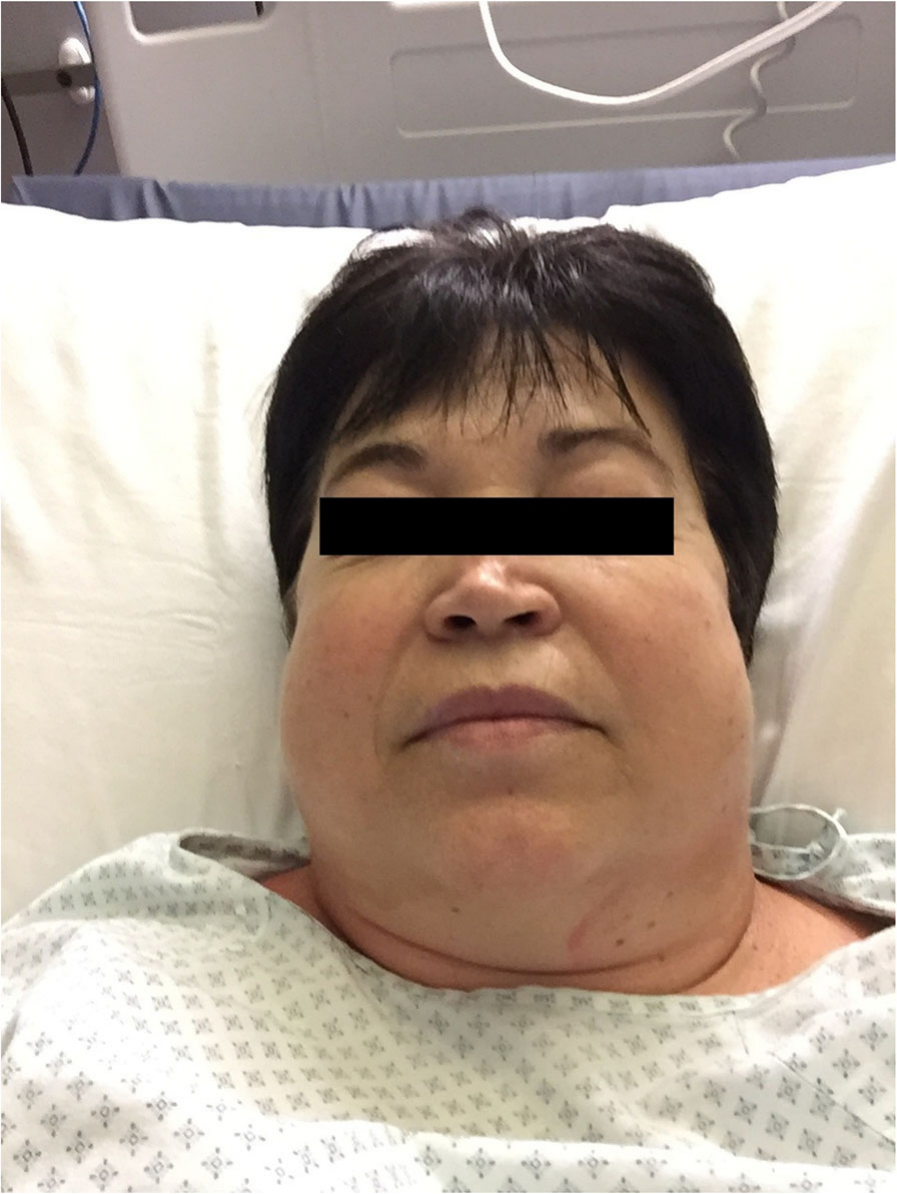
Subcutaneous facial edema

**Fig. 2:**
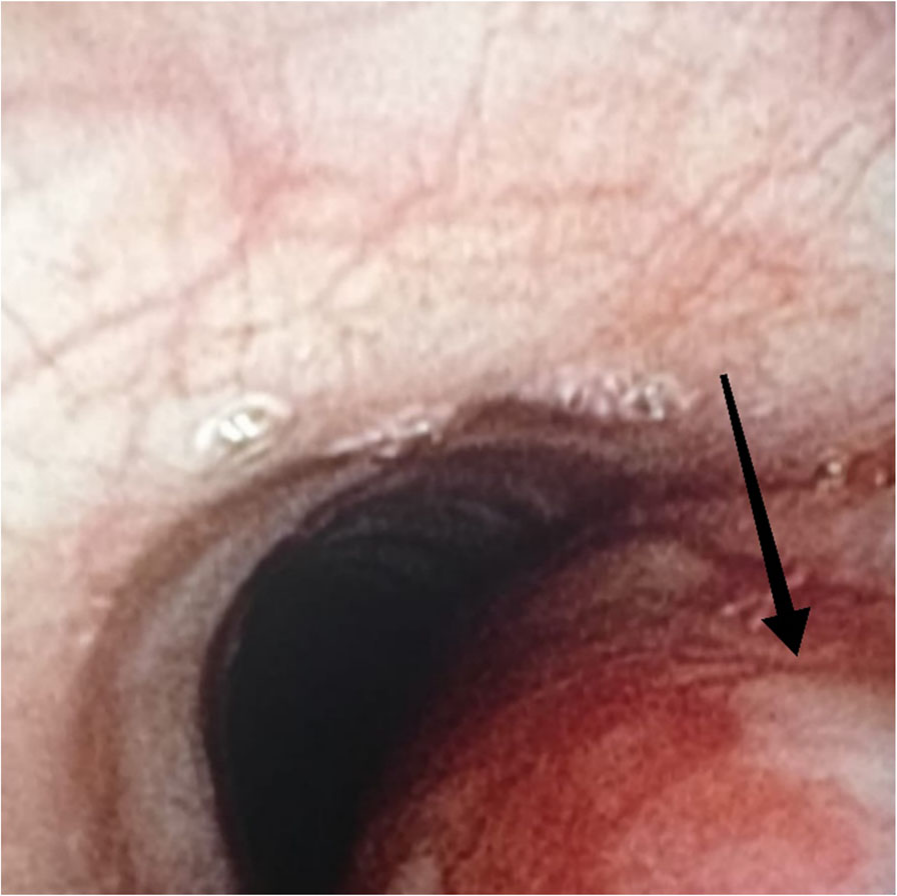
Iatrogenic submucous tracheal rupture, 6 cm below the vocal cords, in the pars membranacea covered with fibrinous tissue that required no further treatment

After 1 h of PACU stay, she was finally discharged onto the surgical ward. The Medical Emergency Team (MET) was called 6 h later because the patient presented latero-cervical subcutaneous emphysema (SE). Vital signs were within the normal range. The physician who visited her noticed left subcutaneous facial edema and mild crackling on palpation in the jugular region (Fig. 1). The patient reported a feeling of swelling; thus, a CT scan of the neck and thorax was performed to rule out the possibility of PNX. The CT scan showed bilateral latero-cervical SE, subcutaneous edema, and signs of PNM. No PNX was found. The patient was reassessed 6 h later on the same day by the same physician, who reported unchanged clinical conditions. Antibiotic treatment was started with amoxicillin/clavulanate 2.2 g every 6 h. The patient underwent a bronchoscopy evaluation the day after, which revealed a likely iatrogenic submucous tracheal rupture, 6 cm below the vocal cords, in the pars membranacea, covered by fibrinous tissue (Fig. 2). No further treatment was required. She was finally discharged home after 5 days in good clinical conditions and without any SE.

## Methods

A systemic review of the literature was performed to collect all case reports of patients undergoing orthopedic shoulder surgery (“population”) and developing complications such as subcutaneous emphysema (SE), PNM, or PNX (“outcome”). Our goal was to compare the risk factors for SE/PNM/PNX for each reported case in order to establish the frequency of one or more risk factors occurring (“comparisons”). In conducting the review, AMSTAR 2 publication standards for systematic reviews were followed [3]. Six databases were screened: PubMed (1996–present), Embase (1974–present), Scopus (2004–present), SpringerLink (1950–present), Ovid Emcare (1995–present), and Google Scholar (2004–present). The following keywords were used: “subcutaneous emphysema,” “pneumomediastinum,” “pneumothorax,” “shoulder surgery,” “intubation,” and “emergency” to search each of the selected databases. Case studies published between 1990 and the present were considered. The literature review was registered on PROSPERO as CRD42021260370.

### Data extraction

Two authors (MD and DO) retrieved the full texts of the relevant articles. All other related titles and abstracts were retrieved and their full versions obtained. The reference lists of the included studies and review articles were manually searched to identify any additional studies relevant to the analysis. Full-text documents were initially assessed for relevance and rapidly assessed using the Critical Appraisal Skills Program (CASP) checklist. Articles that did not meet CASP’s essential criteria (such as relevance to the review aim) were excluded from further analysis.

### Eligibility criteria

Reported cases of SE, PNM, or PNX (including cases involving more than one of these conditions) as complications of orthopedic surgery were included (“intervention”). No age restrictions were considered.

### Exclusion criteria

Studies involving non-human patients, preclinical research cases, research protocols, policy statements, and guidelines were excluded. Urgent interventions due to major trauma and associated with pre-existing pneumothorax of traumatic origin were not taken into account. Case reports on pediatric patients were also excluded.

### Summary of the literature

Given the extreme heterogeneity of the case reports, we considered a quantitative synthesis to be unfeasible. We summarized the evidence from the literature by presenting the results of the individual studies included. We extracted and reported the following data for each of the case studies considered: the study’s identification number, the age and sex of the patient, the reason for endotracheal intubation, the local anesthetic used, the time interval between possible injury and diagnosis, the presenting symptoms, diagnostic tests used, any evidence of tracheal injury and its location, treatments given, and prognosis.

## Results

**Fig. 3:**
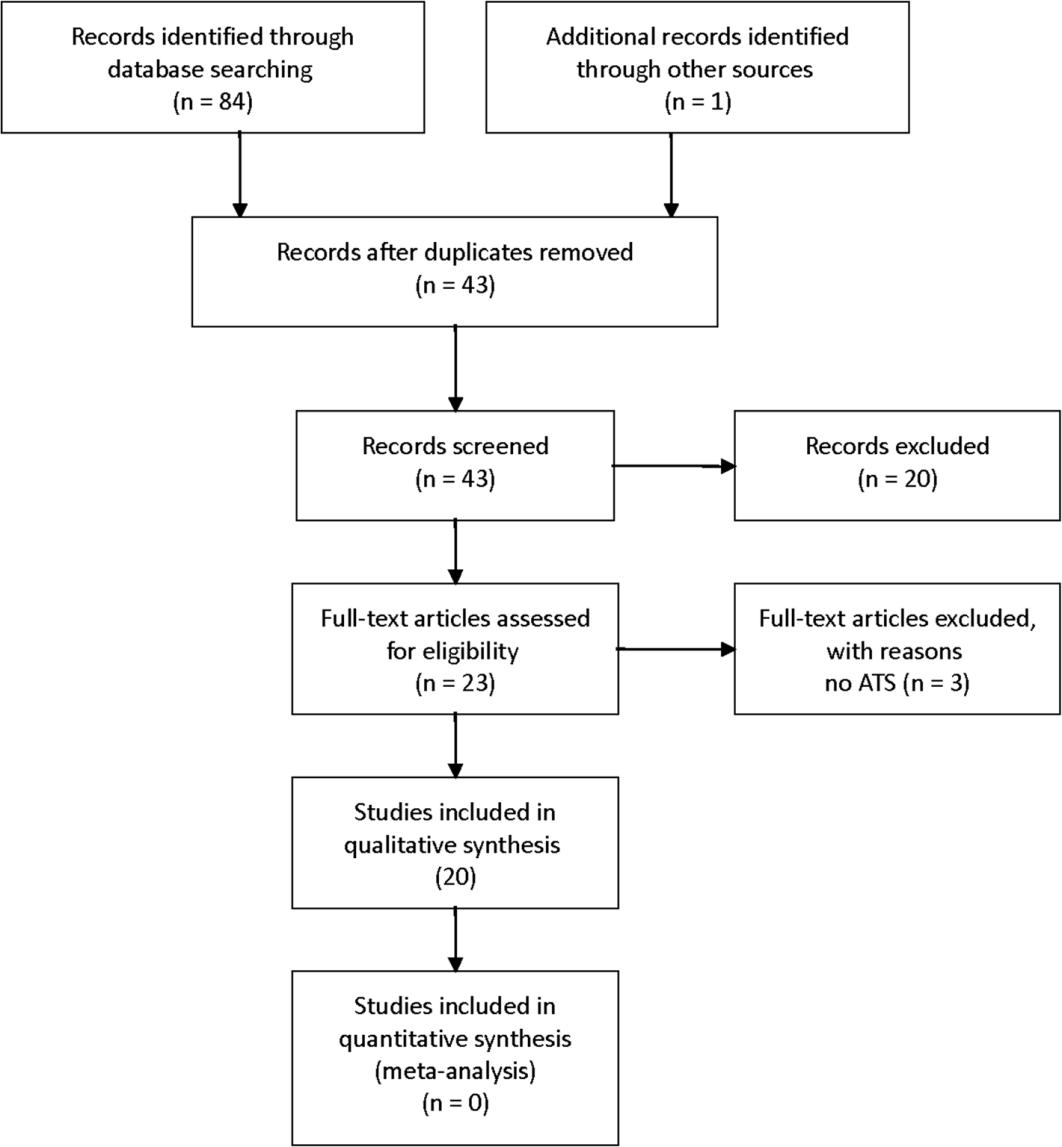
PRISMA flowchart

Twenty-five cases met the eligibility criteria (Fig. 3) [1, 4–20]. In twenty-four cases, the patient underwent general anesthesia and orotracheal intubation; in 9 of these, a plexus anesthetic block was also performed (Table 1). One case involved ATS in the absence of general anesthesia, which was instead performed under plexus anesthetic block only [2]. In 4 cases, the patient was male. Patient age ranged from 22 to 77 years. None of the cases experienced any form of intubation complications undergoing endotracheal intubation. In most cases, the first clinical manifestation (almost always the development of SE in the face or neck or a stinging sensation in the chest) occurred just after the patient had woken up from anesthesia or in the postoperative period. Only in one case did progressive arterial desaturation occur during surgery. In 10 cases, the diagnostic finding was PNM. In 5 cases, the diagnostic finding was associated with PNX. PNX was detected in 17 cases. In 2 cases, SE was found in the absence of any evidence of either PNM or PNX. A tracheal lesion was identified in 3 cases. In all cases, the outcome was benign, and the clinical condition resolved conservatively (without any intervention). Regarding respiratory complications concerning anesthetic techniques, it emerged that SE/PNM after ATS under general anesthesia developed in 3 cases after an average of approximately 160 min, whereas the average time needed to develop PNX was more than double that (542 min). Finally, it was found that in 19 cases, there were reported complications on the operated side (confirmed by radiographic investigation in 14 cases).

**Table 1:** List of case reports included

References	Gender	Age	Reason for IOT	Problems during IOT	ALR (US)	Interval injury-diagnosis (h)	Presenting symptoms	RX/CT /FOB	Evidence of Tracheal injury and its location	Treatment/outcome and discharge (days)
**ATS + ETI**										
**Our case**	F	54	Elective ATS Right shoulder	NO	Posterior SSB (US)	6	Facial edema, SE Laterocervical right > left	RX: bilateral thoracic SE + PNM CT: anterior + Bilateral laterocervical SE + PNM	FOB: Submucous tear in posterior wall for 4.5 cm and 6 cm deeper from vocal cords	Conservative 5 days
Lau [4]	M	63	ATS right shoulder	NO	NO	2	SE on right shoulder, neck, retrosternal pain.	RX: SE + PNM	FOB: no evidence of pharyngeal, laryngeal or tracheal injury	Conservative 7 days
Lee et al. [1] case 2	F	43	ATS right shoulder	NO	NO	Awakening	SE	RX: SE + PNM		Conservative 72h
Sivaram et al. [5]	F	41	ATS left shoulder	NO	NO	4	Severe pain in the chest. SE on left chest and upper eyelid	RX + CT: SE + PNM + bilateral PNX,	CT: intact trachea and bronchi	Conservative 5 days
Bamps et al. [6]	F	42	ATS left shoulder	NO	NO	10	Left chest pain, difficulty breathing, SE on the left shoulder and neck region. Respiratory distress and hemodynamic shock	RX: SE + ipsilateral PNX (shift)		Conservative 4 days
Cassone et al. [7]	F	60	Elective ATS right shoulder	NO	NO	3	Right-sided Facial swelling, voice changes, dyspnea, crepitus on right neck, face and chest.	RX: SE + right PNX (70% shift) s		Conservative 3 days
Asghar et al. [8]	F	51	Elective ATS right shoulder	NO	NO	PO 30 min after	Shortness of breath, dysphonia, dysphagia, skin crepitation on the right side.	CT: right PNM and large PNX, residual air in shoulder joint, SE on right hemithorax extending into bilateral supraclavicular regions, neck, and anteromedial aspect of right arm.		Conservative
Lee et al. [1] case 1	F	45	ATS right shoulder	NO	NO	Awakening	SE	RX +CT: SE + PNM+PNX bilateral (20% right + 50% left)		Conservative 96h
Lee et al. [1] case 3	M	40	ATS left shoulder	NO	NO	Awakening	SE neck	RX: SE + PNX left		Conservative 48h
Dietzel and Ciullo [9] case 1	M	38	ATS left shoulder	NO	NO	24	Left chest pain, Dyspnea	RX: PNX left sided		2 days
Dietzel and Ciullo [9] case 2	F	22	ATS right shoulder	NO	NO	24	Right chest pain	RX: PNX right sided		4 Days
Dietzel and Ciullo [9] case 3	F	37	ATS left shoulder	NO	NO	24	Left chest pain	RX: PNX right sided		2 days
Dietzel and Ciullo [9] case 4	?	34	ATS right shoulder	NO	NO	RR	Desaturation in RR	RX : PNX right sided		
Kim et al. [10]	F	75	ATS right shoulder	NO	NO	Awakening	SE neck and right chest	RX: SE+PNX right sided		Conservative 6 days
Shariyate et al. [11]	F	61	ATS right shoulder	NO	NO	4	SE + dyspnea	RX+CT: SE +PNX right		Conservative 6 days
Knight et al. [12]			ATS shoulder	YES	?	Midsurgery	SE	RX + CT+ FOB: SE + PNM + PNX	FOB: Tracheal wall injury during IOT (exacerbated by boogie)	
Saseendar 2014	F	75	Mumford procedure	NO	SSB (US)	Midsurgery	SE	RX: SE		Conservative 1 day
**ATS+ETI+ISB**										
Bowden et al. [13]	F	30	ATS right assisted acromioclavicular joint reconstruction (fracture)	NO	ISB (US)	PO (?)	No symptoms	RX routine control : incidental finding PNX right + mediastinal shift		Conservative 4 days
Van Nieuwenhuyse et al. [14]	?	74	Elective ATS Right shoulder	no	ISB (US)	PO awakening	Swollen face, voice distorted, SE right thoracic	Rx: SE Right > left CT: SE+ PNM		Conservative
Tandon et al. [15]			ATS	NO	ISB	?	SE	RX: SE		
Tanoubi et al. [16]	F	63	Elective ATS right shoulder	no	ISB (US)	30	Right Shoulder swelling, progressive to the face	Rx (54h PO): bilateral SE+ PNX right + PNM		Conservative
Leander-Olsson et al. [17]	M	72	ATS right shoulder	NO (videolaringoscopy)	ISB (US)	Midsurgery	Intraoperative desaturation	RX: SE CT: PNX		Conservative 2 days
Niu et al. [18]	F	53	ATS left shoulder	NO	ISB (US)	Intraop 2h50’	SE on face neck, chest to bilateral leg	RX: PNX left, SE		5 days
Sharma and Gandhi [19]	F	56	ATS right shoulder	NO	ISB (US)	PO	SE facial, neck, right shoulder extending till left shoulder. Increment of neck circumference			
**ATS + ISB - NO ETI**										
Calvisi 2008	F	52	Elective ATS left shoulder (sitting position)	No IOT	ISB	< 24	Chest pain, subcutaneous enphisema supraclavear fossa and left side of neck	RX+ CT: SE+ PNM in left thoracic and neck region	FOB: Posterolateral tear at T4 CT: suspected tracheal tear	Conservative 3 days

*ETI* endotracheal intubation, *LRA* loco-regional anesthesia, *RX* chest X-ray, *CT* chest CT scan, *FOB* fiberoptic bronchoscopy, *ATS* arthroscopic shoulder surgery, *ISB* interscalene brachial plexus block

## Discussion

It is customary to consider the possible development of SE, PM, or PNX during ATS due to direct pleural damage resulting from a complication of anesthetic block or laceration of the trachea during endotracheal intubation. However, these three pathological entities have also been reported in contexts other than ATS, either caused by tracheal damage—almost always associated with orotracheal intubation—or other causes. Sporadic cases secondary to barotrauma—for example, due to particularly vigorous Valsalva maneuvers, such as during the delivery or use of a laryngeal mask in spontaneous breathing—could induce other pathogenetic processes in addition to the direct pleural damage being considered [21, 22]. Furthermore, tracheal damage does not seem to be due to endotracheal intubation. Some signs of tracheal laceration found during ATS, even in patients not undergoing endotracheal intubation, seem to point toward causes other than endotracheal intubation [2]. In particular, three prime pathogenetic mechanisms are known to result in SE/PNM/PNX: (1) rupture of the parietal pleura, (2) rupture of the visceral pleura, and (3) alveolar rupture [2]. Alveolar rupture is the most common origin of PNX and may result from the rupture of a bleb. Rupture of the visceral pleura is considered a result of airway trauma during intubation. We speculate rupture of the parietal pleura to be the most frequent underlying cause of PNX; rupture may be related to the surgical methodology, to the subacromial distension used in ATS, or as a result of an anesthesia-related complication. The perforation of the parietal pleura during ISB is rare, occurring in approximately 0.2–0.3% of patients [6]. However, the incidence of PNX after ISB (0.2–3%) is substantially higher than that of spontaneous PNX (0.017%). An analysis of ISB alone without endotracheal intubation is not yet possible as only a single case has been reported in the literature to date [2]. To reduce the procedural risks related to ISB, evidence supports the use of suprascapular nerve block for shoulder surgery [23, 24].

SE is a condition in which air infiltrates the subcutaneous tissues. SE due to arthroscopy can be explained by transient changes in negative pressure in the subacromial space (SA) relative to atmospheric pressure. When the SA pressure is lower than atmospheric pressure due to the suction performed to remove debris, air can enter the SA space. The positive pressure of the infusion pump can subsequently push this air into the subcutaneous tissues after turning the suction off and thus cause SE. Air can enter the axillary sheath and extend through the prevertebral space of the neck surrounding the trachea and esophagus, causing PNM. Increased mediastinal pressure due to positive pressure ventilation or during exhalation can cause a rupture of the mediastinal parietal pleura and eventually PNX [14]. Lee et al. proposed an underlying mechanism of PNM/PNX that involves the arthroscopic pump and shaver system [1]. Intermittent pump infusion is typically used to maintain a relatively constant pressure (approximately 50 mm Hg) in the SA space. When the electric arthroscopic shaver is turned on with high intermittent suction, the pump infusion can continuously keep up with the suction and maintain constant pressure. If a transient pressure drop occurs in the SA, it may become negative relative to atmospheric pressure. The authors speculate that this transient pressure drop could cause air to enter the arthroscopic portals. When the electric shaver with suction is turned off, the positive pressure of the infusion pump may push air into the surrounding soft tissues, causing extensive SE. Air can enter the axillary sheath and prevertebral space of the neck, causing PNM. A further increase in mediastinal pressure due to positive pressure ventilation may cause a rupture of the parietal pleura, resulting in PNX.

Calvisi et al. reported a case of SE and PM after ATS [2]; they speculated a Bernoulli effect as the underlying cause, created by the pump, high-suction razor, and outflow cannula, similar to the mechanism proposed by Lee et al. [1].

The extravasation of the infusion fluid during the ATS procedure is another pathogenetic mechanism that can cause SE/PNM/PNX and air embolism, airway edema, and tracheal compression. The recognized risk factors include prolonged surgery, subacromial pathology, large irrigation volumes, increased working pressure, and obesity [25]. Some authors have reported an association between some of these factors, such as irrigation volume and surgical time, and a significant increase in the cuff pressure of the endotracheal tube without increasing the neck circumference [26]. However, only 5 of the 24 ATS case reports considered here investigated the integrity of the trachea through fiberoptic bronchoscopy or CT scan. None of the reported cases collected investigated risk factors of fluid extravasation during ATS.

Furthermore, according to this hypothesis, PNX could represent the end stage of a progression from SE and PNM. The timing of the onset of symptoms may correlate with the extent of the damage according to the sequence of SE → PNM → PNX. About anesthetic techniques, the times to obtain a respiratory complication are longer with endotracheal intubation. Furthermore, the addition of ISB did not increase the incidence of reported events, even if the onset time for complications was significantly earlier (278 vs. 470 min).

In order to confirm the pathogenetic hypothesis related to the ATS technique or ISB as a predisposing factor, additional data not available in the present series case reports would be necessary [1, 2].

In conclusion, the present analysis confirms that endotracheal intubation and loco-regional anesthesia are not the only predisposing risk factors at play in the pathogenesis of SE/PNM/PNX. Rather, multifactorial pathogenesis seems more probable, necessitating that specific attention is paid during ATS to the change in patient position on the operating bed, to any slipping of the endotracheal tube, to patient monitoring whilst under the drapes, and to the cuff pressure. In the future, it will be necessary to focus not only on the diagnosis of these complications but also on diagnostic investigations, such as CT of the neck, or fiberoptic bronchoscopy, aimed at obtaining the most precise information possible on the impact of ATS on the delicate cervicothoracic anatomical structures.

## Data Availability

Data sharing is not applicable to this article as no datasets were generated or analyzed during the current study. All case reports included in the review are available in Table 1.
